# Improving the safety of radiotherapy treatment processes via incident‐driven FMEA feedback loops

**DOI:** 10.1002/acm2.14455

**Published:** 2024-08-05

**Authors:** Dominik Kornek, Michael Lotter, Juliane Szkitsak, Christopher Dürrbeck, Andre Karius, Oliver J. Ott, Carolin Brandl, Christoph Bert

**Affiliations:** ^1^ Department of Radiation Oncology Universitätsklinikum Erlangen, Friedrich‐Alexander‐Universität Erlangen‐Nürnberg (FAU) Erlangen Germany; ^2^ Comprehensive Cancer Center Erlangen‐EMN (CCC ER‐EMN) Erlangen Germany

**Keywords:** error rates, failure mode and effects analysis (FMEA), incident reporting system (IRS), radiotherapy, risk assessment

## Abstract

**Background:**

Failure mode and effects analysis (FMEA) is a valuable tool for radiotherapy risk assessment, yet its outputs might be unreliable due to failures not being identified or due to a lack of accurate error rates.

**Purpose:**

A novel incident reporting system (IRS) linked to an FMEA database was tested and evaluated. The study investigated whether the system was suitable for validating a previously performed analysis and whether it could provide accurate error rates to support the expert occurrence ratings of previously identified failure modes.

**Methods:**

Twenty‐three pre‐identified failure modes of our external beam radiotherapy process, covering the process steps from patient admission to treatment delivery, were proffered on dedicated FMEA feedback and incident reporting terminals generated by the IRS. The clinical setting involved a computed tomography scanner, dosimetry, and five linacs. Incoming reports were used as basis to identify additional failure modes or confirm initial ones. The Kruskal–Wallis *H* test was applied to compare the risk priorities of the retrospective and prospective failure modes. Wald's sequential probability ratio test was used to investigate the correctness of the experts’ occurrence ratings by means of the number of incoming reports.

**Results:**

Over a 15‐month period, 304 reports were submitted. There were 0.005 (confidence interval [CI], 0.0014–0.0082) reported incidents per imaging study and 0.0006 (CI, 0.0003–0.0009) reported incidents per treatment fraction. Sixteen additional failure modes could be identified, and their risk priorities did not differ from those of the initial failure modes (*p* = 0.954). One failure mode occurrence rating could be increased, whereas the other 22 occurrence ratings could not be disproved.

**Conclusions:**

Our approach is suitable for validating FMEAs and deducing additional failure modes on a continual basis. Accurate error rates can only be provided if a sufficient number of reports is available.

## 1 INTRODUCTION

Failure mode and effects analysis (FMEA) is an often used risk assessment method for proactively preventing adverse events in healthcare,^1^ and is increasingly being used for radiotherapy.^2^, ^3^, ^4^, ^5^, ^6^, ^7^, ^8^ To this end, an interprofessional team composed of radiation oncologists, medical physicists, radiation therapists, information technologists, and other professionals involved in the treatment map out a high‐risk therapy process in order to investigate incorrect executions of process steps (failure modes) which may end in adverse events up to harmful failures. The FMEA, among others, thus serves as a decision‐making tool for implementing adequate controls that aim at preventing or detecting failures potentially leading to adverse events.^9^

However, composing an interprofessional team with the required expertise and logistical availability for regular FMEA meetings poses a serious challenge.^10^ Failure mode ratings obtained through expert opinions are necessarily subjective. Furthermore, due to the general complexity of radiotherapy processes, the analysis can take a few weeks to a few months to complete,^11^ and, once completed, the FMEA outputs may not be valid in terms of credibility, comprehensiveness, risk correlation, or risk prediction.^12^, ^13^

The main purpose of this study was to test a novel incident reporting system (IRS) that is linked to the FMEA database. This combined prospective‐retrospective system aimed at remedying the aforementioned problems by involving all personnel by offering them the opportunity to report safety‐relevant clinical issues which could then be investigated through “top‐down” FMEA. In the top‐down approach, failure modes are identified that explicitly result in the incident under consideration. The secondary purpose was to evaluate whether the number of reported incidents potentially affects failure mode occurrence ratings.

## 2 METHODS

### 2.1 IRS

**FIGURE 1:**
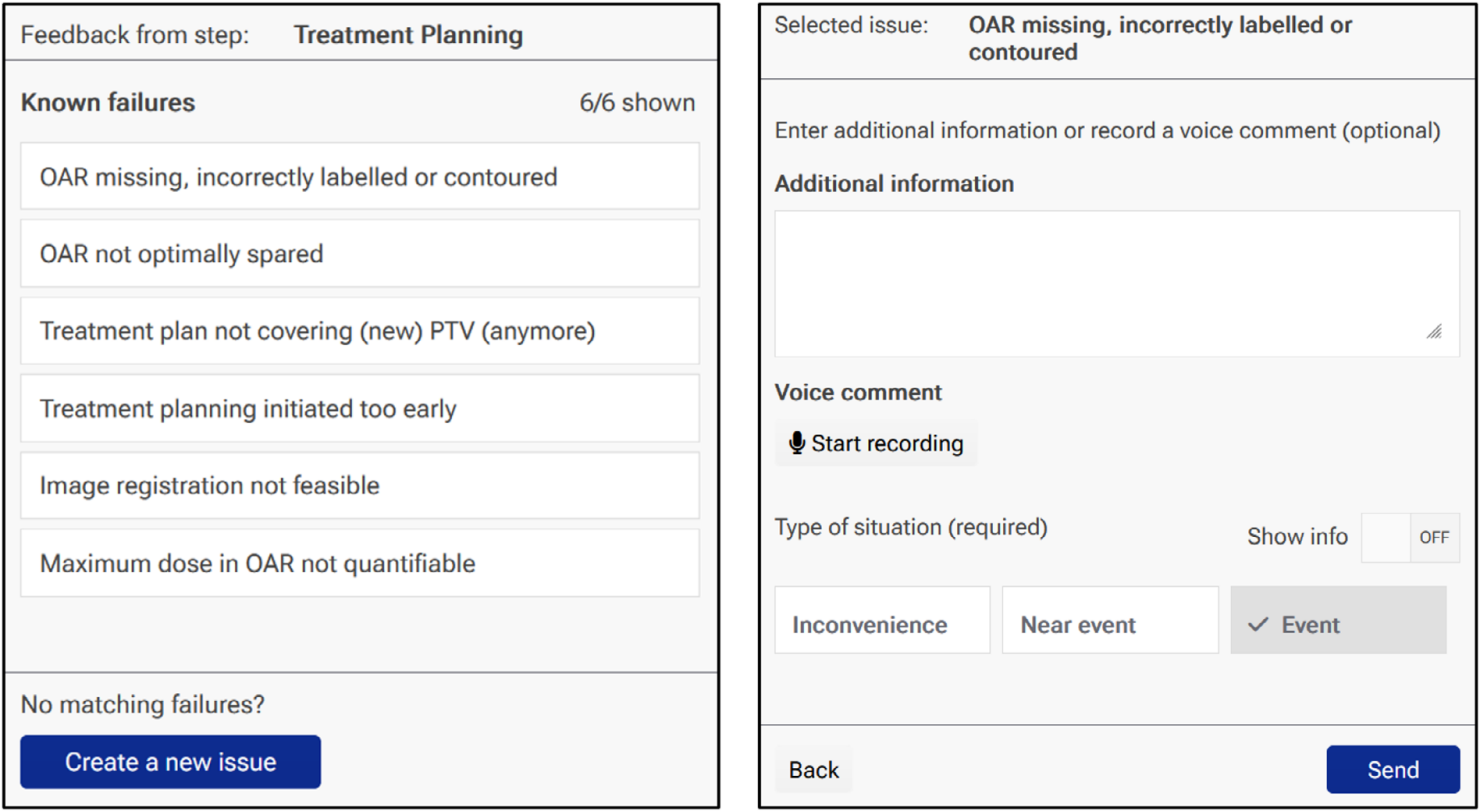
User interface of the failure mode and effects analysis (FMEA) feedback and incident reporting terminal. The terminal is exemplarily shown for the treatment planning process step. The proffered failure modes had been identified previously using the FMEA functionality of the software. Left: failure mode selection, right: further specification of the incident.

For this work, myQA PROactive 2.1.1.0 (IBA Dosimetry GmbH, Schwarzenbruck, Germany) was used. In a recent paper, we described an earlier PROactive (version 1.6.1.0) that facilitated sophisticated FMEA functionality via a web server and its commissioning for clinical use.^14^ In the current version an IRS has been integrated that is linked to the server's FMEA database. Figure 1 shows the user interface of the IRS.

Within our department only, the IRS could be accessed by personnel through feedback terminals (i.e., dedicated web pages) via regular workstations or dedicated handheld devices. Feedback terminals did not require any login to allow anonymous reports and could be generated dynamically for each process step that had been defined in the FMEA's underlying process map. Here, a process step was defined as a station that patients or their associated files pass (e.g., imaging, planning, treatment delivery, etc.). The IRS required two steps for report submission:

1. In the first step (Figure 1, left), a list of buttons (i.e., options) was proffered to the reporter, each reading the string of a different pre‐identified failure mode linked to the respective process step. An additional button reading “create a new issue” was available if no failure mode matched the incident to be reported. Reporters must press a button to continue with the second step.
2. In the second step (Figure 1, right), additional information could voluntarily be input via an unrestricted text box. However, reporters must declare whether the incident to be reported was an *adverse event* (unintended event that has negative influence upon healthcare processes,^15^ such as unintended dose in extremities due to incorrect patient positioning) or a *near‐miss event* (event that may cause an adverse event, but fails to do so because of chance or because it is intercepted,^15^ such as a single incorrect treatment delivery, rectifiable through the following treatment session); if none of these applied, then the incident was classified as an *inconvenience*. This type of incident might refer to a documentation error, repeated imaging studies or similar issues, usually leading to no greater harm than time delays or minor dose deviations. Depending on the selected type of incident, standardized forms were proffered according to our department's policy (e.g., adverse events required, inter alia, the patient's personal information, the reporter's personal information, and the exact event sequence).

Upon report submission, mails were delivered to safety officers, that is, to authorized staff handling potentially confidential reports. The reports were then processed and analyzed as described in Section 2.4.

### 2.2 FMEA data

As mentioned above, myQA PROactive has been commissioned prior to this study, which involved preparing and importing 51 failure modes that had been identified through interprofessional FMEA meetings.^14^, ^16^ Twenty‐three of these were associated with our standard external beam radiotherapy (EBRT) process and relevant for the present study. To pilot test the novel IRS, only feedback terminals for the EBRT process were generated.

### 2.3 Clinical setting

At the time of this study, our department operated one computed tomography scanner (∼55 imaging studies per week) and five linacs (∼940 fractions per week). The IRS has been launched in September 2022. Four handheld devices have been given to radiation oncologists as well as imaging, planning, and treating personnel. All personnel were shown how to use the IRS. Then, in the introduction phase (∼3 months), personnel were asked on a weekly basis to report incidents, regardless of the implied severity. If the incident to be reported matched a failure mode description, the associated button should be pressed; otherwise, a new issue should be opened.

### 2.4 Analysis of reports

Upon mail delivery, both a retrospective and a subsequent prospective analysis of the submitted report were initiated. Safety officers were able to re‐classify reports, for example, if an inconvenience was actually a near‐miss event.

The retrospective track was supervised by two safety officers (a medical physicist and a quality management representative). As the IRS was not clinically implemented but pilot tested, the safety officer's actions were only evolving over time. During this study, they were responsible for timely resolving issues which required interviewing involved personnel, analyzing failed barriers, and proposing new barriers. Moreover, they informed all personnel via mail and bi‐annual in‐house training about the lessons learned in an attempt to raise risk awareness and to prevent the recurrence of incidents. Especially the tasks of interviewing personnel and informing personnel via mail were experimental actions that had not been performed before the launch of the IRS.

One safety officer (another medical physicist) trained in FMEA was responsible for the prospective track. Here, reports describing new issues were firstly triaged with respect to applicability to the EBRT risk assessment. This was the case when reports described error paths that resulted or could result in unintended exposures to radiation. If reports falsely described new issues even though respective failure modes existed in the database, then these reports were linked to those failure modes. If no failure mode applied, then a top‐down FMEA^17^ was performed with the purpose of deducing failure modes that might contribute to the reported event in any way. Failure modes were only deduced for process steps and functions that were involved in the report, which were part of the report data. The results of the retrospective analyses, if available, were considered.

**FIGURE 2:**
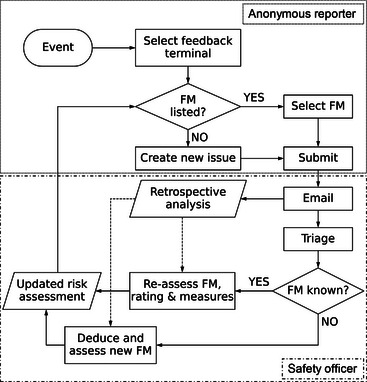
Flowchart of the FMEA feedback loop for iteratively updating the FMEA. FMEA, failure mode and effects analysis; FM, failure mode.

Failure mode ratings for severity (*S*), occurrence (*O*), and detectability (*D*) were suggested by the safety officer using the rating system described in a previous work by Kornek et al.^14^ More precisely, the severity was, just like in the prospective FMEA, assessed in terms of the worst‐case scenario,^14^ independently of the report's outcome. For instance, if a near‐miss event described a potential delivery error, then the severity would refer to the clinical effect had the delivery error occurred during all treatment sessions. The detectability value followed from the quality of the detection controls already in place (e.g., a four‐eye check would correspond to a fixed non‐detection probability *P_det_ *= 10% and, in consequence, *D *= 7).^14^ The occurrence value was proportional to [*d*⋅(1‐*P_det_*)]^−1^, where *d* was the number of days since the launch of the system. All additionally deduced failure modes including their suggested ratings had been reviewed and adjusted in interprofessional FMEA meetings before these changes were accepted and finally added to the EBRT FMEA. This step facilitated that the new failure modes were displayed on the associated feedback terminals and concluded the incident‐driven FMEA feedback loop, as can be seen in Figure 2.

The respective distributions of the risk priority number (RPN) of the initial and the additional failure modes were analyzed by means of the Kruskal–Wallis *H* test, a non‐parametric analysis of variance, to evaluate whether there was a difference in risk priority between prospective and retrospective (incident‐driven) failure modes.

Independent of the analyses, all occurrences of the reported failure modes were counted to perform an ongoing comparison with the expected (Poisson‐distributed) occurrence rates. Wald's sequential probability ratio test^18^ (SPRT) was applied to determine when the respective failure mode occurrence ratings should be updated. This update was designed in a way so that the occurrence numbers were only ever incremented by one on the ten‐step rating system referenced above. Due to the risk of underreporting,^19^ occurrence ratings were never decremented. In other words, the initial occurrence ratings of the experts were treated as the initial minimum occurrence rates. Here, α and β error levels of 0.10 were used to define the type I and II error levels, respectively. The tests were started 3 months after the launch of the IRS in order to consider the potential lack of reports due to the introduction phase.

## 3 RESULTS

Between September 2022 and mid‐December 2023, 304 reports were submitted in total (approx. 19.6 reports per month). Of these, 17 and 34 reports concerned the imaging (3388 imaging studies) and treatment delivery (58,313 fractions) processes, respectively. Therefore, there were 0.005 reported incidents per imaging study and 0.0006 reported incidents per treatment fraction. The confidence intervals, obtained through the variance of the monthly incident rates, were [0.0014, 0.0082] for the imaging process and [0.0003, 0.0009] for the treatment delivery.

**FIGURE 3:**
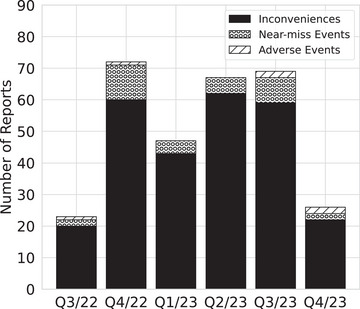
Overview of the reported incidents per quarter, subdivided into adverse events, near‐miss events, and inconveniences. The fourth quarter of 2023 was not complete (2 weeks missing) at the time of this study.

Figure 3 shows the number of reports per quarter, sub‐divided into adverse events, near‐miss events, and inconveniences as they were re‐classified by the safety officers. It can be seen that the IRS has been in constant use since the end of the first month after launch. Fifty‐nine (19.4%) events were retrospectively analyzed (the other events were not further investigated, e.g., because of minor relevance or repetition of inconveniences) and 133 (43.8%) events were relevant for the EBRT FMEA. More specifically, six out of six (100%) adverse events, 31 out of 32 (96.9%) near‐miss events, and 96 out of 266 (36.1%) inconvenient events were pertinent to the prospective analysis. Irrelevant for both analyses were reports describing negligible effects, for example, software or hardware failures, or complaints affecting personnel rather than patients such as time pressure. However, these issues were transferred to and discussed in quality management team meetings.

**FIGURE 4:**
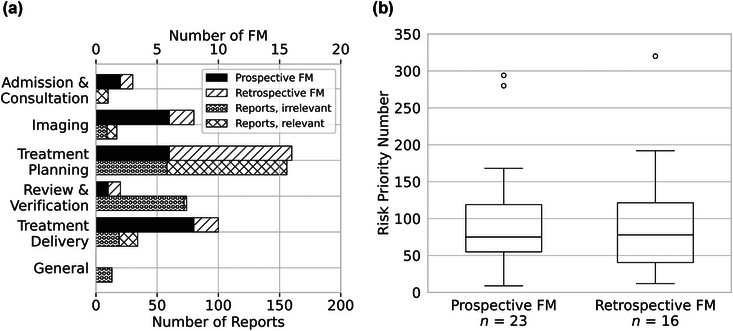
(a) Overview of the initial (prospective) and added (retrospective) FM of the EBRT risk assessment (upper bars) as well as the reports (lower bars), distributed over the respective process steps; (b) Box plots of the RPN distributions of the initial (prospective) and added (retrospective) FM (*p* = 0.954). FM, failure mode; RPN, risk priority number.

Sixty‐five (48.9%), 125 (94.0%), and 40 (30.1%) of the 133 aforementioned reports contained additional information about possible failure causes, failure modes, and failure effects, respectively. Ninety‐one (68.4%) of the reports were linked to 11 pre‐identified failure modes, while the remaining 12 failure modes have not been reported at all. The other 42 reports were considered in the prospective analyses (top‐down FMEA), and as a result, 16 distinct failure modes could additionally be deduced and assessed (see Figure 4a and Table 1). Starting from 23 initial failure modes, therefore, the EBRT risk assessment grew by a factor of 1.7. In addition, we applied the Kruskal–Wallis *H* test to compare the RPNs of the 23 initial and 16 added failure modes. As can be seen in Figure 4b, no significant difference between these two groups (*p* = 0.954) was found.

**TABLE 1:** Sixteen failure modes obtained through the FMEA feedback loop.

	Failure chain			RPN	
Process step – *Function*	Failure effect	Failure mode	Failure cause	*S‐O‐D*	Examples
Admission and consultation – *Administrator creates new patient data set in oncology information system*	Unexpected deterministic effect	Second data set created for the same (pre‐treated) patient	Administrator creates new data set because the original data set was not found as a result of mistyping	320 *8‐5‐8*	Usually, patient data are automatically imported by card reading devices. However, if the administrator enters data manually and mistypes (e.g., birthday), pre‐existing patient data sets will not be found, and a new data set will be created.
Treatment planning – *Physician delineates/contours structures on planning CT*	Severe toxicity or tumor underdosage	PTV contours incorrect	Radiation oncologist contours targets not as prescribed due to miscommunication or mishaps	192 *8‐3‐8*	Contours may be discrepant from the prescription (e.g., missing nodes), or the boost volume is larger than the primary target, or there are partial contours in random slices, and so forth
Treatment planning – *Physician documents radiotherapy prescription*	Unexpected deterministic effect	Treatment plan planned with incorrect prescription	Radiation oncologist documents (pre‐irradiated and) planned dose incorrectly due to mistyping or miscommunication	144 *8‐3‐6*	If the previous dose is documented as 50.4 Gy instead of 59.4 Gy, then the current plan will be planned with more dose than intended.
Treatment planning – *Physician delineates/contours structures on planning CT*	Moderate toxicity	Dose levels for different targets incorrectly documented	Radiation oncologist documents different dose levels incorrectly due to miscommunication	126 *7‐3‐6*	Upon changing the treatment concept from a sequential to an integrated boost, dose levels need to be adapted.
Review and verification – *Planner exports set‐up fields to additional system*	Plan mix‐up	PTV label in additional systems (e.g., ExacTrac) incorrect	Planner transfers PTV labels incorrectly due to mix‐ups or previously saved information	120 *8‐3‐5*	The oncology information system and the X‐ray monitoring system use independent databases, requiring that PTV labels in both systems are identical. If they are not, metastasis A might be imaged and treated with an incorrect plan, thinking A is metastasis B.
Treatment delivery – *Therapist selects intended course/plan in record and verify system or treatment unit database*	Plan mix‐up	Incorrect plan applied	Therapist refers to duplicate patient file or treatment protocol which has been (re‐) created due to oversight	112 *8‐2‐7*	If a second patient file and treatment protocol are created when there should be only one, mix‐ups of plans might happen.
Treatment planning – *Model contours target(s) (CTV, GTV) on planning CT*	Severe toxicity	Normal tissue incorrectly contoured as target volume (e.g., bleeding as metastasis)	Deep‐learning model (for research purposes) auto‐contours incorrectly due to unhealthy normal tissue appearing malign	112 *8‐2‐7*	Trained auto contouring model detects metastasis on a follow‐up CT scan which has been treated and controlled before. Therefore, every detected metastasis should be double checked to avoid treating them again.
Treatment planning – *Physician delineates/contours structures on planning CT*	Moderate toxicity	Structure set for incorrect CT study created	Radiation oncologist mixes‐up different CT study sets (e.g., expiration vs. free breathing)	84 *7‐3‐4*	Structures are contoured in one study (e.g., expiration), and after a treatment concept change, copied into another study (e.g., free breathing) without adapting them.
Treatment planning – *Planner inserts treatment couch model*	Mild toxicity or tumor underdosage	Treatment plan does not consider treatment couch	Planner omits adding the treatment couch	72 *6‐2‐6*	Treatment couches must be added manually on CT scans, which might have gone forgotten.
Treatment planning – *Physician delineates/contours structures on planning CT*	Unexpected deterministic effect	Cardiac pacemaker missing, incorrectly labelled or contoured	Radiation oncologist creates another structure instead of using the pre‐defined structure	60 *5‐3‐4*	Radiation oncologist might oversee the pre‐defined structure and creates another one, thereby rendering conditional planning scripts ineffective.
Simulation and imaging – *Therapist selects imaging protocol*	Treatment delay	Incorrect imaging protocol applied	Therapist selects incorrect protocol due to large anatomical overlap or other similarities	56 *2‐4‐7*	Instead of using a protocol for the thoracic spine to detect bone metastases, a lung protocol is used.
Treatment planning – *Therapist schedules treatment course*	Treatment delay	Treatment plan finished too late	Therapist schedules day of first treatment session when the CT scan is done rather than when the contours are finished	42 *3‐7‐2*	If contours are not finished in time, then delays might be conveyed to the next process step(s).
Simulation and imaging – *Therapist adjusts field of view prior to the CT scan*	Treatment delay	Field of view too small/large	Therapist selects a smaller/larger field of view due to inexperience or unclear instructions	36 *2‐3‐6*	
Treatment planning – Planner labels treatment fields	Plan mix‐up	Treatment fields labelled ambiguously	Planner labels fields other than specified in the pertaining standard operating procedure	32 *8‐2‐2*	PTVs and corresponding fields follow a specific format: A, B, C, and so on for each consecutive treatment course. If the fifth PTV is, again, labelled “D”, then PTVs might be mixed up.
Treatment delivery – Patient validation system validates patient in real‐time	Patient mix up	Beam clearance revoked too late	Patient validation system, which interlocks the beam clearance, clocks at a frequency not high enough	30 *5‐1‐6*	When the patient validation system refreshes, for example, every 15 seconds, it might be possible to start treatment of the next patient before the beam clearance is revoked. This failure mode could occur during fast IMRT QA measurements which would have no clinical consequence.
Treatment planning – *Physician delineates/contours structures on planning CT*	Treatment delay	PTV missing or incorrectly labelled (e.g., labelled ambiguously)	Radiation oncologist labels different versions of a PTV ambiguously	12 *3‐4‐1*	If two radiation oncologists contour the same PTV, it might be unclear which one to use.

Abbreviations: CT, computed tomography; D, detectability; FMEA, failure mode and effects analysis; O, occurrence; PTV, planning target volume; RPN, risk priority number; S, severity.

**FIGURE 5:**
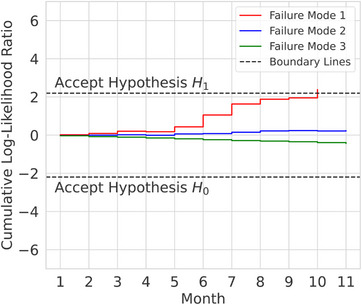
Sequential probability ratio test (SPRT) plots of the three most reported failure modes. Failure modes 1, 2, and 3 were reported 68, 11, and seven times, respectively.

Finally, we applied Wald's SPRT to conclude whether the number of reports could be used to validate the experts’ occurrence ratings. In Figure 5, the SPRT plots of the three most reported failure modes can be seen. More specifically, failure mode 1 refers to “*organ‐at‐risk missing, incorrectly labelled or contoured”* with 68 reports; failure mode 2 refers to “*PTV contours incorrect (e.g., contours discrepant from prescription (e.g., missing nodes), boost larger than primary target, partial contour in random slice, etc.)”* with 11 reports; and failure mode 3 refers to “*second data set created for the same (pre‐treated) patient”* with seven reports. The first failure mode was identified 14 times as a near‐miss event because in these cases, critical organs‐at‐risk contours located in the high dose area would be missing; the other 54 times, the missing organs‐at risks were in low dose areas and therefore, the reports were classified as inconveniences. The second failure mode was re‐classified as a near‐miss event once (the boosted gross tumor volume [GTV] would not encompass the initial GTV), while the others remained inconveniences. And lastly, the third failure mode would be classified as near‐miss events all seven times, as previous treatments could have gone unnoticed and no standardized measure existed that could identify redundant patient data sets.

The null hypotheses *H_0_* were defined as the experts’ occurrence rating of the failure modes equal to the number of reports of the respective failure modes. The alternative hypotheses *H_1_* were defined as the experts’ occurrence rating increased by one, that is, the next higher step on the rating system. According to Figure 5, it was possible after 10 months to show that failure mode 1 should have an occurrence rating of at least *O* = 4 (once a month) rather than *O* = 3 (several times a year), which was the occurrence rating of the experts (*H_0_*). However, for both the other failure modes seen, and, in fact, for the other 36 remaining failure modes, the alternative hypotheses were never accepted due to the low numbers of reports per failure mode. As the RPNs of these 38 failure modes remained unchanged, the currently implemented risk mitigation measures associated with these failure modes were still considered sufficient. However, failure mode 1 with 68 reports, whose RPN rose by 33%, was mitigated through more refined standard operating procedures, adapted structure set templates and training.

## 4 DISCUSSION

In radiotherapy, there is little observational data available to deduce occurrence and detection probabilities.^8^ Risk‐informed decisions or quality management programs are therefore, often, based on experience and subjectivity, rather than evidence. As stated in AAPM TG‐100,^8^ actual clinical data like observed error rates and reports of near‐miss events and adverse events could be used to inform FMEAs. In this study, we pilot tested a combined prospective‐retrospective system – which is linked to an FMEA database and proffers failure modes on its reporting interfaces – for more than 15 months with the aim to learn from such clinical data and hence increase the informational content of FMEAs. The system was easy to use. Furthermore, by means of incoming reports, analysts were provided input for discussing weaknesses about their FMEA and identifying latent failure modes overlooked during the initial analysis. As a result, prioritization could be improved, and 16 additional failure modes could be identified.

The methodology of extending and validating an existing FMEA by means of the software greatly differs from creating the FMEA in the first place. When conducting a prospective FMEA, usually an inductive or “bottom‐up” method is applied, where a small team of experts maps out the process under consideration and identifies, for each process step, what could fail and how it could fail.^8^ This method is described in great detail in AAPM TG‐100 and is also implemented in myQA PROactive, as has been described in our previous publication.^14^ Here, all reporting personnel contributed to the FMEA, either by confirming proffered failure modes, or by reporting new issues, which could then be analyzed. Therefore, retrospective failure modes were not deduced *en masse* as they are during FMEA team meetings – rather, their deduction was distributed over time and the interval of the FMEA meetings could be lowered. By involving all personnel, more field expertise could be extracted and, thus, the content value of the FMEA could be increased, leading to a more robust quality management program.

Thirty‐eight adverse or near‐miss events were reported through the IRS. Whereas five of six events were identified through the initial FMEA, only 17 of the 31 relevant near‐miss events were identified. This is similar to the results of Yang et al.,^13^ who identified 13 of 33 near‐miss events through their FMEA. However, in contrast to Yang et al.^13^ as well as Gilmore and Rowbottom's^4^ experience, we could not confirm that retrospective failure modes are also, in general, of lower criticality. This may be due to different triaging strategies. In fact, 56.2% of the reports described negligible effects, and failure modes with lower severities could have been deduced, which has not been done here to keep the FMEA practical. Yet, these lower severity reports were analyzed in terms of general process and quality improvement, comparable to the methodology of Mutic et al.^20^

The secondary aim of this study was to show whether the number of reports could be used to verify occurrence ratings. Firstly, 12 initial failure modes have not been reported at all. This could indicate (a) failure modes whose occurrence ratings were so low that further sampling is necessary, (b) failure modes whose prevention measures are simply working as intended, or (c) underreporting.^19^ Whereas (a) is probably true for eight failure modes whose ratings were *O* ≤ 2 (once a year or less), we cannot differentiate between (b) and (c) with our data. However, compared to the IRS experiences of Smith et al.,^21^ who observed 1.3 reported errors per 1000 treatment attendances, our 0.0006 reported incidents per fraction seem too low. Therefore, reporting should further be encouraged in our facility.

Secondly, concerning the failure modes which actually have been reported (*n* = 11), their respective counts were so low that only one failure mode's expert occurrence rating could be disproved (Figure 5). It should be stated here that whether the *H_0_* or *H_1_* hypotheses were accepted in Wald's SPRT, we interpreted the respective occurrence ratings as the *minimum* occurrence ratings rather than the *true* ones. This way, the experts’ ratings were ensured to be protected against underreporting. Considering the possibility of underreporting as well as the low statistics per failure mode, verifying occurrence ratings via local incident reporting did not seem feasible. Especially if there were more initial failure modes – other authors identified up to 361 failure modes^22^ – the statistics would be even worse. Also, in our experience, there were reports that could be linked to several failure modes, there were reports summarizing past failures (e.g., a report would state that the failure mode in question happened a few times before), and there were identical reports submitted by two different reporters. These issues further distorted the statistics. In the future, other methods should be investigated for verifying occurrence ratings. A retrospective approach that automatically queries the number of past success events could be more reliable than the number of sporadic reports. For instance, the number of times structure sets have been finished in time is a straightforward database query, whereas each time a structure set has been finished too late, a separate report is required. This approach is also potentially able to decrement occurrence ratings.

A similar approach that combined IRS and FMEA was described by Paradis et al.^23^ They concluded that actionable results could be identified without a complete FMEA. We agree with their conclusion, however, the value of the initial FMEA should not be neglected, as the analysis’ main purpose is to prevent events proactively.

In this tested version 2.1.1.0 of myQA PROactive, each feedback terminal was linked to a specific process of a specific FMEA. For example, at any linac operation room, the feedback terminals would proffer the failure modes of the treatment delivery process documented in the general EBRT FMEA. However, if there are FMEAs dealing with special EBRT treatment techniques (stereotactic treatment, whole body irradiation, total skin electron irradiation, to name a few), then these FMEAs would require additional physical feedback terminals to display special failure modes that can also occur at the linac, which is unpractical. We also found that if there were too many failure modes displayed, then in order to save time, reporters would simply skip the failure mode selection and submit a “new issue”. In future versions, an improved concept is needed to keep reporting simple and user‐friendly while proffering all possible failure modes. This could be implemented through a live analysis of the event to be reported, potentially using a trained linguistic model that pre‐filters eligible failure modes across FMEAs.

Another limitation of this study is that within the tested clinical setting, more than 60 radiation oncologists, medical physicists, and therapists could voluntarily report, and a continuous stream of reports has been developed. In smaller departments with less staff, we expect the number of reports to naturally be lower, so the usefulness of our approach remains to be shown for these departments.

## 5 CONCLUSION

Our pilot study showed that the safety of our EBRT process could be improved with a combined prospective‐retrospective approach by identifying latent failure modes “on‐the‐fly”. The recorded events were also useful for identifying additional causes for pre‐identified failure modes as well as process improvements beyond the scope of FMEA. Further methods to validate failure mode ratings should be investigated in the future. Then, expert assessments are needed in order to decide whether the software is to be implemented into clinical routine. Even though this study focused on EBRT, it is equally feasible to cover other radiotherapy services such as brachytherapy, intraoperative radiotherapy, and so forth with our approach.

## AUTHOR CONTRIBUTIONS

**Dominik Kornek**: Conceptualization; methodology; software; formal analysis; investigation; writing—original draft; visualization. **Michael Lotter**: Methodology; validation; writing—review & editing. **Juliane Szkitsak**: Investigation; writing—review & editing. **Christopher Dürrbeck**: Investigation; writing—review & editing. **Andre Karius**: Writing—review & editing. **Oliver J. Ott**: Validation. **Carolin Brandl**: Methodology; validation. **Christoph Bert**: Methodology; validation; writing—review & editing; supervision.

## CONFLICT OF INTEREST STATEMENT

Dominik Kornek and Christoph Bert have a patent pending on some functionality of myQA PROactive. Michael Lotter, Juliane Szkitsak, Christopher Dürrbeck, Andre Karius, Oliver J. Ott, and Carolin Brandl have no relevant conflicts of interest to disclose.

## References

[1] Liu HC , Zhang LJ , Ping YJ , Wang L . Failure mode and effects analysis for proactive healthcare risk evaluation: a systematic literature review. J Eval Clin Pract. 2020;26(4):1320‐1337. doi:10.1111/jep.13317 31849153

[2] Bright M , Foster RD , Hampton CJ , Ruiz J , Moeller B . Failure modes and effects analysis for surface‐guided DIBH breast radiotherapy. J Appl Clin Med Phys. 2022;23(4):e13541. doi:10.1002/acm2.13541 35112445 PMC8992938

[3] Rassiah P , Su FF , Huang YJ , et al. Using failure mode and effects analysis (FMEA) to generate an initial plan check checklist for improved safety in radiation treatment. J Appl Clin Med Phys. 2020;21(8):83‐91. doi:10.1002/acm2.12918 PMC7484852 32583912

[4] Gilmore MDF , Rowbottom CG . Evaluation of failure modes and effect analysis for routine risk assessment of lung radiotherapy at a UK center. J Appl Clin Med Phys. 2021;22(5):36‐47. doi:10.1002/acm2.13238 PMC8130239 33835698

[5] Gray T , Antolak A , Ahmed S , et al. Implementing failure mode and effect analysis to improve the safety of volumetric modulated arc therapy for total body irradiation. Med Phys. 2023;50(7):4092‐4104. doi:10.1002/mp.16466 37265031

[6] Esposito M , Mancosu P , Bruschi A , et al. Correction to: the role of EPID in vivo dosimetry in the risk management of stereotactic lung treatments. Strahlenther Onkol. 2024;200(1):106. doi:10.1007/s00066-023-02168-5 37923943

[7] Baehr A , Hummel D , Gauer T , et al. Risk management patterns in radiation oncology‐results of a national survey within the framework of the Patient Safety in German Radiation Oncology (PaSaGeRO) project. Strahlentherapie Und Onkologie. 2022;199(4):350‐359. doi:10.1007/s00066-022-01984-5 35931889 PMC10033570

[8] Huq MS , Fraass BA , Dunscombe PB , et al. The report of Task Group 100 of the AAPM: application of risk analysis methods to radiation therapy quality management. Med Phys. 2016;43(7):4209. doi:10.1118/1.4947547 27370140 PMC4985013

[9] Commission E. RADIATION PROTECTION N° 181. General guidelines on risk management in external beam radiotherapy; 2015.

[10] Ashley L , Armitage G , Neary M , Hollingsworth G . A practical guide to failure mode and effects analysis in health care: making the most of the team and its meetings. Jt Comm J Qual Patient Saf. 2010;36(8):351‐358. doi:10.1016/s1553-7250(10)36053-3 20860241

[11] Wegener S , Exner F , Weick S , et al. Prospective risk analysis of the online‐adaptive artificial intelligence‐driven workflow using the Ethos treatment system. Z Med Phys. 2022. doi:10.1016/j.zemedi.2022.11.004 PMC11384068 36504142

[12] Shebl NA , Franklin BD , Barber N . Failure mode and effects analysis outputs: are they valid? BMC Health Serv Res. 2012;12:150. doi:10.1186/1472-6963-12-150 22682433 PMC3405478

[13] Yang F , Cao N , Young L , et al. Validating FMEA output against incident learning data: a study in stereotactic body radiation therapy. Med Phys. 2015;42(6):2777‐2785. doi:10.1118/1.4919440 26127030

[14] Kornek D , Menichelli D , Leske J , et al. Development and clinical implementation of a digital system for risk assessments for radiation therapy. Z Med Phys. 2023. doi:10.1016/j.zemedi.2023.08.003 PMC11384085 37666699

[15] DIN EN 15224:2017‐05. Quality management systems—EN ISO 9001:2015 for healthcare; German version EN 15224:2016. Berlin: Beuth Verlag; 2017. doi:10.31030/2581525

[16] Lohmann D , Lang‐Welzenbach M , Feldberger L , et al. Risk analysis for radiotherapy at the Universitatsklinikum Erlangen. Z Med Phys. 2022;32(3):273‐282. doi:10.1016/j.zemedi.2021.11.002 35012863 PMC9948825

[17] IEC 60812:2018. Failure modes and effects analysis (FMEA and FMECA). Berlin: Beuth Verlag; 2018.

[18] Wald A . Sequential tests of statistical hypotheses. The Ann Math Stat. 1945;16(2): 117‐186, 70.

[19] Kessels‐Habraken M , Van der Schaaf T , De Jonge J , Rutte C , Kerkvliet K . Integration of prospective and retrospective methods for risk analysis in hospitals. Int J Qual Health Care. 2009;21(6):427‐432. doi:10.1093/intqhc/mzp043 19828551

[20] Mutic S , Brame RS , Oddiraju S , et al. Event (error and near‐miss) reporting and learning system for process improvement in radiation oncology. Med Phys. 2010;37(9):5027‐5036. doi:10.1118/1.3471377 20964222

[21] Smith S , Wallis A , King O , et al. Quality management in radiation therapy: a 15 year review of incident reporting in two integrated cancer centres. Tech Innov Patient Support Radiat Oncol. 2020;14:15‐20. doi:10.1016/j.tipsro.2020.02.001 32181375 PMC7063337

[22] Ibanez‐Rosello B , Bautista JA , Bonaque J , et al. Failure modes and effects analysis of total skin electron irradiation technique. Clin Transl Oncol. 2018;20(3):330‐365. doi:10.1007/s12094-017-1721-3 28779421

[23] Paradis KC , Naheedy KW , Matuszak MM , Kashani R , Burger P , Moran JM . The fusion of incident learning and failure mode and effects analysis for data‐driven patient safety improvements. Pract Radiat Oncol. 2021;11(1):e106‐e113. doi:10.1016/j.prro.2020.02.015 32201319

